# Evaluation of Social Media Addiction and Its Relationship with Anxiety and Academic Performance Among Medical and Non-Medical Students: A Cross-Sectional Study from Saudi Arabia

**DOI:** 10.3390/healthcare13030295

**Published:** 2025-01-31

**Authors:** Suhaj Abdulsalim, Mohammed Saif Anaam, Maryam Farooqui, Mohammed S. Alshammari, Saeed Alfadly, Jehad Alolayan, Anas Ahmad Aljarallah, Saud Alsahali

**Affiliations:** 1 Department of Pharmacy Practice, College of Pharmacy, Qassim University, Buraydah 52571, Saudi Arabia; m.anaam@qu.edu.sa (M.S.A.); s.alfadly@qu.edu.sa (S.A.); jehad.s.olayan@gmail.com (J.A.); s.alsahali@qu.edu.sa (S.A.); 2 Discipline of Social and Administrative Pharmacy (DSAP), School of Pharmaceutical Sciences, Universiti Sains Malaysia (USM), Gelugor 11800, Pulau Pinang, Malaysia; m.farooqui@usm.my; 3 Department of Pharmacy Practice, College of Pharmacy, University of Hafr Albatin, Hafr Albatin 39524, Saudi Arabia; moalshammari@uhb.edu.sa; 4 Mouwasat Hospital, Abi Jafar Al Mansour St. Garnatah, Riyadh 13241, Saudi Arabia; anasaljarallah6@gmail.com

**Keywords:** social media addiction, anxiety, academic performance, medical and non-medical students, Saudi Arabia, cross-sectional study

## Abstract

Background: Social media (SM) use has become an integral aspect of daily life. Overutilization of SM can adversely impact an individual’s physical and emotional well-being, especially that of students. This study evaluated the potential impact of SM addiction on anxiety and academic performance among university students. Methods: A cross-sectional study was conducted among medical ad non-medical students at Qassim University during September 2022–April 2023 after obtaining the Ethics Committee’s permission. Raosoft was used for calculating the sample size, and participants were selected through convenience sampling. Both descriptive and inferential statistics were used to analyze and interpret the results, using SPSS version 25. Results: A total of 269 students participated in the study. The majority of them were male (52%), with a mean age of 22.28. The main purpose of SM usage was entertainment, followed by communication. More than 30% of them were using SM for 4–6 h per day, accessing SM 1–10 times in a day, with more than half of them feeling that they had SM addiction and that it was affecting their daily activities and sleep. The majority of them agreed that SM can be used for group discussion (78.1% vs. 71.6%) and knowledge sharing (93.7% vs. 90%). However, a statistically significant difference was observed about anxiety level between the two groups. A negative correlation was found between cumulative grade point average (CGPA) and anxiety level. Conclusion: The findings suggest that SM has both positive and negative effects on academic performance and social anxiety. Continuous education and motivation about wise use of SM is warranted among students by parents, university authorities, and policymakers.

## 1. Introduction

In recent decades, the widespread use of social media (SM) has increased exponentially. Although the majority of people utilize SM platforms for connecting with others, networking, and obtaining information, a minority of users develop an addiction to social networking sites, leading to compulsive or excessive usage [1,2]. According to research, between 5 and 10% of Americans qualify as being addicted to SM [3]. Social media addiction (SMA) exhibits excessive preoccupation with SM, uncontrollable urges to access it, and interference with other significant aspects of one’s life [3,4].

SMA operates similarly to substance addiction, with individuals experiencing mood changes, obsessive preoccupation, increasing tolerance, and withdrawal symptoms [5]. This means that addicted individuals may feel a sense of pleasure or positive emotional changes when they use SM, become fixated on it, need to use it more frequently or for longer periods to achieve the desired effect, and experience unpleasant physical or emotional symptoms when they try to stop using it. Consequently, after a period of abstinence, addicted users may resume their excessive SM utilization.

SM can lead to both physical and psychological dependency because of its impact on the brain. Sharing personal information on social networking platforms activates the same brain areas as taking addictive drugs. The reward region of the brain influences both choices and emotions through its chemical signaling pathways. When a person participates in gratifying activities or consumes something addictive, dopamine levels rise due to the activation of dopamine-producing neurons in the brain. Consequently, the brain receives a “reward” and associates the behavior or substance with positive reinforcement [6].

As of October 2024, the number of active SM users reached nearly 5.22 billion [7]. Those who are addicted to SM often experience emotional distress when denied access to these platforms [8]. Behavioral addiction manifests itself through six primary factors, including salience, tolerance, mood alteration, relapse, withdrawal, and conflict [9]. Being aware of the symptoms and ramifications of addiction is crucial in promoting healthy SM use. Therefore, it is essential to practice moderation, balance online activity with other aspects of life, and prioritize one’s overall well-being [10]. Studies have provided evidence that overreliance on SM has been linked to issues such as anxiety, depression, cyberbullying, and poor sleep habits in young individuals [11,12]. These negative impacts can have severe and lasting effects on an individual’s physical and emotional well-being, highlighting the need for responsible use of SM. SM platforms have been shown to have a considerable impact on individuals’ mental health, both positively and negatively. Therefore, it is crucial for individuals to appreciate the benefits of these platforms, while also recognizing the need for moderation and balanced usage [13]. Invisible and potentially harmful impacts of excessive SM use include poor sleep, a drop in academic performance, and unpleasant emotional outcomes including anxiety and sadness [14]. Most previous studies about SMA relied on surveys and questionnaires for assessing behavioral addiction, lacking a complete clinical diagnosis. Considered to be most susceptible to problematic internet use are adolescents and students [15].

SM is now becoming an integral part of users’ lives, particularly those of young adults and students. Undergraduates use SM more than the general population, and among them, particularly those studying medicine and paramedicine. WhatsApp and Facebook are the most popular platforms [16]. Medical students can experience significant psychological distress due to their demanding academic responsibilities. Additionally, those transitioning from high school to medical school may encounter numerous challenges and engage in risky behaviors, such as addiction, depression, difficulty identifying emotions, and anxiety. If today’s students struggle with an addiction to SM, it may impede their ability to perform daily activities and hinder their learning experience [16]. In students’ lives, SM usage averages about 1–3 h in a day, and they are using it for networking, socializing, entertainment, and information. Interestingly, students use SM for completing their academic activities such as assignments, projects, and for communication with their peers and instructors [17]. Overusage of SM can negatively impact the executive functions of a student’s life [18].

The impact of SMA, which is very prevalent in academics, shows a correlation between addiction and anxiety [19]. Anxiety can impact all aspects of life, and academic performance is especially prone to anxiety. Previous studies have reported that anxiety is very much connected with addiction, whether it is SM, smoking, or using different kinds of drugs [13,20]. An editorial in 2020 reported that internet addiction and SM usage is very high in the female student population compared to the male population in Saudi Arabia [21]. Although the culture of Saudi Arabia restricts females to being in indoors more compared to males, recent developments are allowing females more latitude in terms of outdoor activities. Although there are few studies from Saudi Arabia about internet addiction and its impact on academic performance from different regions, only one study has been reported from the Qassim region on the impact of SMA on the academic performance of university students [22]. The present study hypothesized that there is a significant positive relationship between SM addiction and anxiety levels and a negative association with academic performance among students. Hence, we undertook this study to evaluate the impact of SMA on anxiety and academic performance among medical and non-medical students.

## 2. Materials and Methods

### 2.1. Study Design and Participants

A cross-sectional study was conducted among medical and non-medical students at Qassim University after obtaining the Ethics Committee’s permission from the Deanship of Scientific Research, Qassim University during September 2022–April 2023. We followed a convenience sampling method. Both male and female students from medical and non-medical colleges who were willing to participate were included in the study. Students other than those at Qassim University, and incomplete questionnaires, were excluded from the study.

### 2.2. Study Questionnaire Development and Administration

A self-administered, validated questionnaire was adapted from a previous study [17]. Five sections were included in the final questionnaire. Participants’ demographics in the first section were followed by pattern of SM usage in the second section. In the third section, impact of SM usage was assessed. Perception about SM usage was assessed in the fourth section. In the fifth section, a social anxiety scale [23] was used. The final questionnaire was translated into Arabic for clear understanding and a better response from students. A validation process was conducted by obtaining the opinion of experts like academicians and linguistic experts. The final version was tested by a pilot study on a sample of 10 students to check clarity, understanding, and ease of use. To confirm the validity of the translated questionnaire a back translation process was conducted by linguistic experts. The English and Arabic versions of the questionnaire were distributed among university students through Google Forms. The link was sent to all the class leaders and shared through the class WhatsApp group to obtain the maximum reach. Two reminders were sent every 2 weeks to obtain the maximum response from students. Cronbach’s alpha was used to test the reliability and validity of the questionnaires and was found to be 0.827 for perception about SM usage and 0.942 for the social anxiety scale, which is acceptable as per the literature.

### 2.3. Sample Size

Using Raosoft software [24], the sample size was calculated. Considering a 95% confidence level and 5% margin of error, with a 50% response rate, for a population of 850 medical and non-medical students, the sample size was estimated to be 265 students.

### 2.4. Ethical Considerations

This research was conducted on humans according to the guidelines of the Declaration of Helsinki after obtaining the Ethics Committee’s permission (23-41-20) from the Deanship of Scientific Research, Qassim University (Buraydah, Saudi Arabia).

### 2.5. Data Analysis

For data entry and analysis, Statistical Package for Social Sciences (SPSS) version 25 was used. Descriptive analysis was used for the study. For inferential analysis, Chi-square was used to find the association between level of anxiety of students and SMA. Moreover, to find the correlation between cumulative grade point average (CGPA) and anxiety level among medical and non-medical students, a two-sample *t*-test was used. A *p*-value of < 0.05 was considered as significant.

## 3. Results

**Figure 1:**
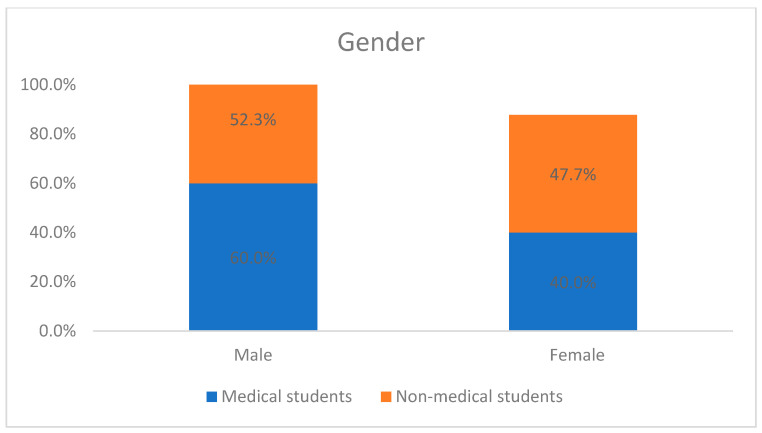
Gender distribution of the study participants.

**Figure 2:**
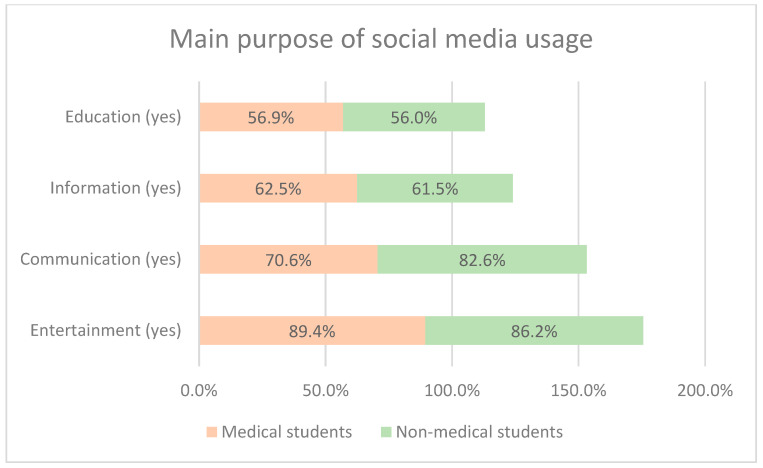
Main purpose of SM usage (multiple answers were allowed).

A total of 269 students completed the survey, out of which 160 (59.5%) were medical students and 109 (40.5%) were non-medical students. Males were dominant in both groups (60% in medical vs. 52.3% in non-medical). Mean age was 22.34 ± 1.79 and 22.22 ± 1.97 (medical vs. non-medical). The favorite place for SM usage was found to be home for both groups (100%), followed by university (96.9% vs. 86.2%), with *p*-value = 0.001. The majority of the students from both groups agreed that entertainment (89.4 vs. 86.2) was the main purpose of SM usage, followed by communication (70.6 vs. 82.6). Most of them agreed that a mobile phone was used for accessing SM (100% vs. 96.3%), and they were using multiple SM platforms. Facebook (97.5% vs. 95.4%) and Snapchat (73.8% vs. 65.1%) were the favorite platforms. The details of the demographics of the study participants are given in Table 1 and Figure 1 and Figure 2.

### 3.1. Pattern of SM Usage Among Study Participants

**Figure 3:**
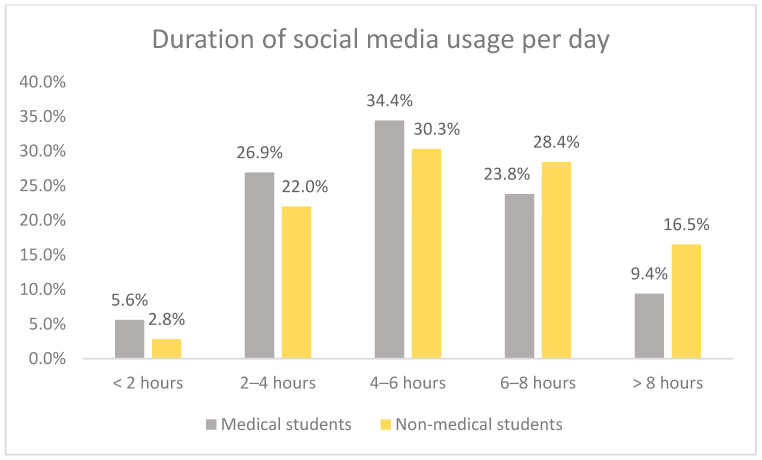
Duration of SM usage per day.

Most study participants, comprising medical and non-medical students, agreed that they use SM for 4 to 6 h per day (34.4% vs. 30.3%). The predominant time for SM usage was reported to be in the evening for both groups (70% vs. 51.4%), with a *p*-value = 0.002. More than half of the participants indicated that they check their SM accounts 1 to 10 times a day. Additionally, many agreed that they access SM whenever they have spare time (50% vs. 61.5%). Details of the patterns of SM usage are provided in Table 2 and Figure 3.

### 3.2. Impact of SM Usage in Daily Life

**Figure 4:**
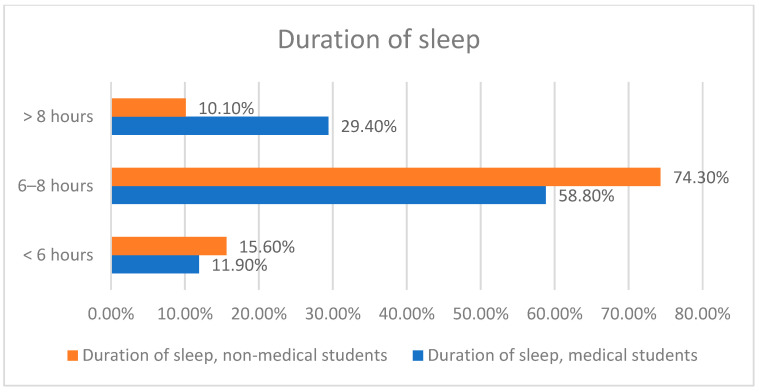
Duration of sleep. Data analyzed by Chi-square; *p* = 0.001 *.

Many participants agreed that they use SM immediately after waking up (76.9% vs. 67.9%) and just before going to sleep (89.4% vs. 86.2%). The majority of them expressed their willingness to use SM platforms for academic purposes (79.4% vs. 84.4%). Additionally, more than half reported feeling that they have developed SMA (58.1% vs. 53.2%). Over one-third indicated that SM usage is affecting their health (41.9% vs. 36.7%), while two-thirds acknowledged that it has an impact on their daily activities (71.9% vs. 68.8%). Approximately 70% of study participants agreed that SM usage affects their study time (66.9% vs. 73.4%). The majority also reported sleeping for 6 to 8 h a day (58.8% vs. 74.3%), although most indicated experiencing sleep problems at times (66.2% vs. 74.3%). Details regarding the impact of SM usage among study participants are provided in Table 3 and Figure 4.

### 3.3. Perceptions About SM Usage

More than two-thirds of students from both medical and non-medical groups agreed that social networking tools enhance creativity and interactivity (70.1% vs. 66%). A significant majority indicated that they could actively participate in group discussions when using SM (78.1% vs. 71.6%). Additionally, most students felt that they could personalize their learning through SM applications (70.6% vs. 75.2%). About 90% agreed that social networking tools facilitate knowledge sharing (93.7% vs. 90%), and that SM networks help them access information easily (83.1% vs. 93.5%). However, over one-third of the students expressed neutral feelings when asked whether using SM improved study habits (37.5% vs. 34.9%). Furthermore, many agreed that SM sites enhance interaction with classmates and lecturers (50% vs. 63.3%), assist in completing assignments (88.1% vs. 94.5%), and enable the students to be knowledge producers rather than solely consumers (53.1% vs. 67%). A statistically significant difference (*p* < 0.05) was found in statements 1, 5, 6, 7, and 9 between the two groups. Details regarding perceptions of SM usage are provided in Table 4.

### 3.4. Social Anxiety Scale for SM Usage

Approximately 34.0% of medical students reported rarely feeling anxious about their actions on SM that others might find awkward, while 25% stated they never felt anxious about their online behavior. In contrast, among non-medical students, 48.6% revealed that they never felt anxious about their actions on SM, and 25.7% reported feeling anxious only rarely. A statistically significant difference (*p* = 0.001) was observed in anxiety levels between the medical and non-medical groups. When asked about their concern regarding disapproval of their behavior by others, a statistically significant difference (*p* = 0.001) was also found between the two groups, with 34.4% of medical students and 58.7% of non-medical students answering that they never felt concerned. Details regarding the social anxiety scale for SM usage are provided in Table 5.

### 3.5. Correlations Between CGPA and Anxiety Scores

The Pearson correlation test showed a statistically significant association between CGPA and anxiety scores (*p* < 0.05). The highest correlation coefficient was observed among non-medical students (r = −0.287), followed by medical students, where the correlation coefficient was r = −0.253. The negative correlation suggests that as CGPA increases, the total anxiety score tends to decrease in both groups (Table 6).

#### 3.5.1. CGPA

To compare the mean CGPA scores between medical and non-medical students, a two-sample *t*-test was performed. No statistically significant difference was found in CGPA scores between medical students (M = [3.79], SD = [0.79]) and non-medical students (M = [3.74], SD = [0.76]); *t* (267) = [0.57], *p* = [0.57].

#### 3.5.2. Total Anxiety Score (TAS)

To compare the mean TAS scores between medical and non-medical students, a two-sample *t*-test was performed. A statistically significant difference was found in the TAS scores between medical students (M = [50.1], SD = [16.8]) and non-medical students (M = [43.0], SD = [17.6]); *t* (267) = [3.34], *p* = [0.001].

## 4. Discussion

SM plays an important role in our daily lives. SM is used for various purposes including connecting people, communication, exchange of information, etc. Although SM provides students with several learning opportunities, such as digital literacy, communication skills, community building, and socialization, its detrimental effects on students’ lives cannot be overlooked. Overuse of SM may lead to academic distraction, cyberbullying, sleep disruption, addiction, and overdependence. The potential for addiction arises from the fact that SM can be highly stimulating, providing instant gratification and a sense of social validation.

In Saudi Arabia, university students’ SMA is a major concern [25]. The high usage rate of SM among students in this age group makes them particularly susceptible to addiction, which leads to adverse consequences for their academic performance and mental health. Although there has been a previous study from the Qassim region [22], this study explores the impact of SMA on anxiety and academic performance among medical and non-medical students.

The majority of our study participants were male (60%), which is consistent with the findings of an Iranian study [19] and a Turkish study [26]. They found that SMA is based on the time they spend on their mobile phones in a day. Globally, gender differences are evident in certain internet use behaviors and disorders, where males exhibit higher rates of internet gaming disorder (IGD) and females demonstrate higher levels of SMA [27]. The mean age of our study sample was approximately 22 years, similar to previous studies from Saudi Arabia [16,25], where most participants (57% and 59%) were in the age groups of 21–23 and 20–24, respectively. SMA statistics [3] from the United States indicate that adults aged 18–22 constitute 40% of Americans addicted to SM.

In the present study, the main purpose of SM usage was entertainment (> 80%), followed by communication (> 75%) and information (> 60%). These percentages were higher compared to another study by Alsaud et al. (2019) which reported 58%, 69%, and 75%, respectively [28]. The observed variation may be attributed to the fact that the later study was conducted only among female students, suggesting that females may be more interested in searching for information rather than using SM solely for entertainment or communication.

All our study participants (100%) have an account on WhatsApp, followed by Snapchat (70%). This is in line with a previous study from Abha, Saudi Arabia [16] which reported 90% for WhatsApp and 84% for Snapchat. Al Suwairi et al. (2016) reported 90% for WhatsApp and 87% for Snapchat, while Al Saud et al. (2019) reported 78% for WhatsApp and 85% for Snapchat [28,29]. In contrast to our results, Al Shalawi found that Snapchat was the most frequently used SM platform in his study [30]. However, authors also agree that WhatsApp was used for academic purposes more frequently than other social networking platforms. The variation may be due to regional differences and variations in student populations. As a number of accounts on different SM platforms consumes more time for each youngster, they are losing time for academic activities. Our findings align with a study by Chandrasena et al., which confirms that the number of SM accounts plays a major role in daily activities, sleep, and study time [17].

The duration of SM usage plays a major role in a person’s behavioral, academic, and social development, particularly among the student population. In the present study, 32% of participants reported using SM for 4 to 6 h per day. This is higher than the global average of 2 h and 27 min per day [31]. Our findings align with a previous study by Alshanqiti et al., where 49.5% of the participants used SM for 3 to 5 h per day [32]. Additionally, two other studies reported 1 to 3 h of SM usage among medical students [33,34]. Interestingly, studies from Turkey and Indonesia revealed that SM usage increased after the COVID-19 pandemic [35,36]. This may be attributed to the shift to online courses and classes in the post-pandemic period, resulting in higher usage of SM and the internet.

In contrast, research conducted in Bahrain found that the majority of participants logged in more than 20 times a day [37]. In the present study, however, over half of the participants used SM every day, with a frequency ranging from 1 to 10 times per day, consistent with findings from other studies [38,39]. The majority of participants were shown to use their preferred SM platforms whenever they had free time. This is comparable to other studies [17,40], which unequivocally show that students use SM whenever it is convenient for them. However, if they are using it during college hours, their usage should be limited to academic purposes only.

The impact of SM on daily life was also assessed in the present study. It has been evident that more than 80% of our participants were using SM either before sleeping or immediately after waking up. This kind of behavior is pointing to how SM influences students’ lives. This is alarming, and university policymakers should act accordingly to create awareness about the safe and wise use of SM among students. These activities must be strictly controlled in order to prevent issues such as anxiety, depression, social distraction, etc. Interestingly, we found that about 80% of our study participants agreed that they are willing to use SM for academic purposes. This positive attitude should be encouraged. Perceived SMA was reported in more than half of the study participants in the present study. Moreover, our participants agreed that SM has an effect on health, daily activities, and study time. This is in line with a Sri Lankan study, where they reported that use of SM can impact study time and daily activities [17]. This is alarming, and it is necessary to counsel and convince the youngsters about the wise use of SM.

In the present study, the average CGPA was found to be 3.8, which is higher than in a U.S. study (3.54) [41] and another Saudi Arabian study (3.2) [30], but lower than in another study (4.29) [16]. Although the age of participants was similar in these studies, academic performance has been found to be different in the present study. This could be attributed to the fact that the U.S. study included interdisciplinary students, while Mansour et al. focused solely on medical students. Moreover, Alshalawi reported a positive correlation between SM use and academic performance [30]. However, those who were addicted to SM also reported having depression and anxiety [20]. In contrast, some studies [42,43,44,45,46] reported a negative correlation between SM usage and academic performance, while others [47,48,49] found no relationship at all. Hence, it can be concluded that SM usage may impact academic performance if students do not use it wisely.

Social anxiety scale assessment in the present study population clearly indicates that SM usage can lead to social anxiety in the majority of participants. A statistically significant association was found between CGPA and the anxiety score (*p* < 0.05), highest among non-medical students. Moreover, a negative correlation was found between CGPA and the anxiety level of participants. To be precise, when CGPA increases, the anxiety level decreases. Similarly, a Malaysian study reported that there is a negative correlation between SMA and CGPA [50]. The results suggest that SMA is influencing students’ mental health and academic performance. A Jordanian study also revealed that SMA has a direct effect on stress and anxiety level of students, and that it indirectly impacts academic performance [51]. This is supported by a recent systematic review by Abbouti S. et al., and another bibliometric analysis about SMA and academic performance and a few studies have reported that stress can be managed by being addicted to SMA [20,52,53,54]. These results highlight the importance of addressing SMA among university students in Saudi Arabia through education and awareness programs, early screening, and effective preventive measures such as creating awareness about the hazards of SMA, one-to-one counselling, etc.

Hazardous effects of SMA can be minimized by creation of awareness programs at college level, university level, or country level by the Ministry of Education and policymakers to allow the students to lead their best academic and personal lives. Furthermore, parents and families also need to recognize the potential harms of SM overuse in their children. With the widespread availability of smartphones and internet access, children and teenagers are becoming increasingly reliant on SM platforms for entertainment, socialization, and information gathering. Parents can play an important role in educating their children about responsible SM usage and setting reasonable limits on their usage.

This study possesses certain limitations that are typically found in cross-sectional surveys, such as the challenge of establishing causal connections based on the analyses conducted. Additionally, the research focused solely on students registered at Qassim University, limiting the generalizability of the findings to the broader population of students attending universities in other cities throughout Saudi Arabia. We cannot overlook the possibility of confounding variables’ effects on the results. Lastly, the use of a convenience sampling method along with a self-report scale could have potentially introduced biases such as reporting and recall bias that may affect the accuracy and interpretation of the results.

## 5. Conclusions

SMA is a growing concern among university students in Saudi Arabia, and its negative impacts extend beyond just mental health to academic performance and overall well-being. The findings suggest that SMA can have significant consequences for students’ mental health and academic performance. It is, therefore, imperative that universities and educators recognize the potential harms of excessive SM usage and implement measures to prevent and address addiction among their student populations. Effective and targeted interventions, including education and awareness programs, screening, and early detection and treatment, are critical to address the issue of SMA among university student populations. Nonetheless, it is important to recognize that SM also has many positive benefits, and therefore a balanced approach is recommended to ensure the safe and responsible use of these platforms for the betterment of society.

## Figures and Tables

**Table 1:** Demographic details of the study participants.

Characteristics	Medical Students (N = 160, n (%))	Non-Medical Students (N = 109, n (%))	*p*-Value
Age mean ± SD	22.34 ± 1.79	22.22 ± 1.97	0.595
CGPA mean ± SD	3.89 ± 0.82	3.88 ± 0.77	0.921
Favorite place of SM usage (multiple answers were allowed)			
Home (yes)	160 (100)	109 (100)	
University (no)	155 (96.9)	94 (86.2)	0.001 *
Café (no)	103 (64.4)	85 (78)	0.017 *
Devices used for SM access (multiple answers were allowed)			
Mobile phone (yes)	160 (100)	105 (96.3)	0.015 *
Tablet (yes)	26 (16.2)	13 (11.9)	0.323
Desktop (yes)	16 (10)	12 (11)	0.790
Laptop (yes)	15 (9.4)	14 (12.8)	0.368
Types of SM accounts (multiple answers were allowed)			
Facebook (no)	156 (97.5)	104 (95.4)	0.350
Snapchat (yes)	118 (73.8)	71 (65.1)	0.129
Twitter (yes)	108 (67.5)	72 (66.1)	0.805
TikTok (yes)	103 (64.4)	66 (60.6)	0.524
Instagram (yes)	90 (56.2)	61 (56)	0.963

* Statistically significant; data analyzed by Chi-square. SM = social media.

**Table 2:** Pattern of social media usage among study participants.

Characteristics	Medical Students (N = 160, n (%))	Non-Medical Students (N = 109, n (%))	*p*-Value
Dominant time for SM usage			
Morning	4 (2.5)	5 (4.6)	0.002 *
Afternoon	21 (13.1)	12 (11)	
Evening	112 (70)	56 (51.4)	
Midnight	23 (14.4)	36 (33)	
Frequency of following SM			
Not daily	16 (10)	6 (5.5)	0.332
1–10 times a day	90 (56.2)	60 (55)	
> 10 times a day	54 (33.8)	43 (39.4)	
Accessing SM			
During free time	70 (43.8)	40 (36.7)	0.079
Any spare time	80 (50)	67 (61.5)	
While at university	0	1 (0.9)	
During meal times	9 (5.6)	1 (0.9)	
During lectures	1 (0.6)	0	

* Statistically significant; data analyzed by Chi-square. SM = social media.

**Table 3:** Impact of SM usage in daily life.

Statements	Medical Students (N = 160, n (%))	Non-Medical Students (N = 109, n (%))	*p*-Value
Use of SM just after waking up (yes)	123 (76.9)	74 (67.9)	0.228
Use of SM just before sleep (yes)	143 (89.4)	94 (86.2)	0.607
Willingness to use SM for academic purposes (yes)	127 (79.4)	92 (84.4)	0.002 *
Perceived SMA (yes)	93 (58.1)	58 (53.2)	0.699
Does SM effect your health? (yes)	67 (41.9)	40 (36.7)	0.394
Does SM have an effect on activities of daily living? (yes)	115 (71.9)	75 (68.8)	0.588
Does SM have an effect on study time? (yes)	107 (66.9)	80 (73.4)	0.254
Presence of sleep problems			
Never	26 (16.2)	20 (18.3)	0.056
Sometimes	106 (66.2)	81 (74.3)	
Always	28 (17.5)	8 (7.3)	

* Statistically significant; data analyzed by Chi-square.

**Table 4:** Perceptions about SM usage.

Statement No.	Statements	Medical Students (N = 160, n (%))					Non-Medical Students (N = 109, n (%))					*p*-Value
		SA	A	N	D	SD	SA	A	N	D	SD	
1	Social networking tools increase students’ creativity and interactivity.	54 (33.8)	58 (36.3)	29 (18.1)	19 (11.9)	0	29 (26.6)	43 (39.4)	32 (29.4)	5 (4.6)	0	0.033 *
2	I can freely create and participate in group discussions through SM.	51 (31.9)	74 (46.2)	28 (17.5)	7 (4.4)	0	40 (36.7)	38 (34.9)	21 (19.3)	10 (9.2)	0	0.176
3	Students will be able to personalize their learning by using the social networking application of e-learning.	56 (35)	65 (40.6)	37 (23.1)	2 (1.2)	0	29 (26.6)	53 (48.6)	25 (22.9)	2 (1.8)	0	0.468
4	Social networking tools facilitate knowledge sharing.	82 (51.2)	68 (42.5)	7 (4.4)	3 (1.9)	0	49 (45)	49 (45)	8 (7.3)	3 (2.8)	0	0.601
5	I can get the information that I want through SM.	85 (53.1)	48 (30)	8 (5)	15 (9.4)	4 (2.5)	65 (59.6)	37 (33.9)	7 (6.4)	0	0	0.007 *
6	Using social networking sites improves my study habits.	21 (13.1)	21 (13.1)	60 (37.5)	39 (24.4)	19 (11.9)	8 (7.3)	30 (27.5)	38 (34.9)	26 (23.9)	7 (6.4)	0.024 *
7	Using social networking sites improves my interaction with classmates and lecturers.	37 (23.1)	43 (26.9)	51 (31.9)	12 (7.5)	17 (10.6)	28 (25.7)	41 (37.6)	20 (18.3)	18 (16.5)	2 (1.8)	0.001 *
8	Social networking sites enables to contact friends/classmates for doing assignments.	82 (51.2)	59 (36.9)	7 (4.4)	11 (6.9)	1 (0.6)	62 (56.9)	41 (37.6)	3 (2.8)	3 (2.8)	0	0.455
9	SM enable me to be a knowledge producer rather than a consumer.	45 (28.1)	40 (25)	59 (36.9)	15 (9.4)	1 (0.6)	22 (20.2)	41 (37.6)	32 (29.4)	9 (8.3)	5 (4.6)	0.029 *

SA = Strongly Agree; A = Agree; N = Neutral; D = Disagree; SD = Strongly Disagree. * Statistically significant; data analyzed by Chi-square.

**Table 5:** Social anxiety scale for social media usage.

	Statements	Medical students, (N=160, n (%))					Non- medical students, (N=109, n (%))					*p*-Value
No		Always	Often	Sometimes	Rarely	Never	Always	Often	Sometimes	Rarely	Never	
1	I feel anxious about the fact that others might find my actions awkward.	7(4.4)	6(3.8)	53(33.1)	54(33.8)	40(25)	1(0.9)	7(6.4)	20(18.3)	28(25.7)	53(48.6)	0.001*
2	I am concerned about being ridiculed by others for the content I have shared.	9(5.6)	11(6.9)	37(23.1)	57(35.6)	46(28.8)	1(0.9)	11(10.1)	17(15.6)	32(29.4)	48(44)	0.020*
3	I am concerned about the fact that the content I share will not be liked by others	10(6.2)	10 (6.2)	28(17.5)	43(26.9)	69(43.1)	4(3.7)	8(7.3)	15(13.8)	30(27.5)	52(47.7)	0.768
4	I am afraid that my close friends will not approve my behavior.	3(1.9)	7(4.4)	37(23.1)	28(17.5)	85(53.1)	2(1.8)	11(10.1)	12(11)	20(18.3)	64(58.7)	0.067
5	I would feel uncomfortable when my friends publicly express their dislike about content I have shared.	11(6.9)	22(13.8)	32(20)	30(18.8)	65(40.6)	5(4.6)	9(8.3)	23(21.1)	30(27.5)	42(38.5)	0.329
6	I am concerned about disapproval of my behaviors by others	8(5)	12(7.5)	36(22.5)	49(30.6)	55(34.4)	1(0.9)	5(4.6)	20(18.3)	19(17.4)	64(58.7)	0.001*
7	I am concerned about being judged about my shared content by my friends in the presence of others.	8(5)	9(5.6)	53(33.1)	28(17.5)	62(38.8)	3(2.8)	4(3.7)	24(22)	21(19.3)	57(52.3)	0.141
8	The possibility of having my private information acquired by others makes me feel anxious.	30(18.8)	28(17.5)	45(28.1)	15(9.4)	42(26.2)	13(11.9)	14(12.8)	22(20.2)	16(14.7)	44(40.4)	0.040*
9	The possibility of having my private information shared publicly makes me anxious.	29(18.1)	32(20)	54(33.8)	15(9.4)	30(18.8)	16(14.7)	7(6.4)	25(22.9)	32(29.4)	29(26.6)	0.001*
10	I would be concerned if my personal space is accessed without my consent	56(35)	32(20)	48(30)	6(3.8)	18(11.2)	30(27.5)	23(21.1)	11(10.1)	16(14.7)	29(26.6)	0.001*
11	I feel anxious about how social media companies/executives handle privacy policy regarding my private life.	28(17.5)	16(10)	44(27.5)	40(25)	32(20)	12(11)	18(16.5)	33(30.3)	15(13.8)	31(28.4)	0.038*
12	I feel anxious when talking with people I have just met.	19(11.9)	16(10)	41(25.6)	30(18.8)	54(33.38)	8(7.3)	10(9.2)	11(10.1)	26(23.9)	54(49.5)	0.006*
13	I feel nervous when I talk with people I do not know very well.	13(8.1)	26(16.2)	45(28.1)	34(21.2)	42(26.2)	7(6.4)	14(12.8)	21(19.3)	23(21.1)	44(40.4)	0.143
14	I feel uneasy while making new friends.	25(15.6)	16(10)	34(21.2)	56(35)	29(18.1)	8(7.3)	12(11)	30(27.5)	12(11)	47(43.1)	0.001*
15	I feel tense when I meet someone for the first time.	26(16.2)	15(9.4)	28(17.5)	61(38.1)	30(18.8)	11(10.1)	8(7.3)	23(21.1)	19(17.4)	48(44)	0.001*
16	I am afraid of interacting with others.	8(5)	18(11.2)	49(30.6)	34(21.2)	51(31.9)	3(2.8)	6(5.5)	20(18.3)	19(17.4)	61(56)	0.002*
17	I feel nervous when I have to talk with others about myself	19(11.9)	13(8.1)	37(23.1)	54(33.8)	37(23.1)	11(10.1)	3(2.8)	15(13.8)	30(27.5)	50(45.9)	0.002*
18	I feel anxious about making a negative impression on people.	27(16.9)	21(13.1)	43(26.9)	43(26.9)	26(16.2)	16(14.7)	3(2.8)	24(22)	29(26.6)	37(33.9)	0.002*
19	I am concerned about people thinking poorly of me.	14(8.8)	18(11.2)	21(13.1)	56(35)	51(31.9)	9(8.3)	7(6.4)	18(16.5)	20(18.3)	55(50.5)	0.007*
20	I feel anxious about not being able to meet people’s expectations.	11(6.9)	12(7.5)	24(15)	37(23.1)	76(47.5)	9(8.3)	6(5.5)	22(20.2)	17(15.6)	55(50.5)	0.475

* Statistically significant, data *Statistically significant, data analyzed by Chi-square.

**Table 6:** Correlations between CGPA and anxiety scores.

		Medical Students (N = 160)		Non-Medical Students (N = 109)	
		CGPA	Total Anxiety Score	CGPA	Total Anxiety Score
CGPA	Pearson Correlation	1	−0.253 **	1	−0.287 **
	Sig. (two-tailed)		0.001		0.002
	N	160	160	109	109
Total Anxiety Score	Pearson Correlation	−0.253 **	1	−0.287 **	1
	Sig. (two-tailed)	0.001		0.002	
	N	160	160	109	109

** Correlation is significant at less than 0.05 level (two-tailed).

## Data Availability

Data will be available upon reasonable request.

## Abbreviations

The following abbreviations are used in this manuscript:

CGPA	Cumulative grade point average
COVID-19	Coronavirus disease 2019
SM	Social media
SMA	Social media addiction
SPSS	Statistical Package for Social Sciences
